# Surface-engineered vertically-aligned ZnO nanorod for sensitive non-enzymatic electrochemical monitoring of cholesterol

**DOI:** 10.1016/j.heliyon.2024.e37847

**Published:** 2024-09-11

**Authors:** Rafiq Ahmad, Kiesar Sideeq Bhat, Vandana Nagal, Umesh T. Nakate, Akil Ahmad, Mohammed B. Alshammari, Shamshad Alam, Byeong-Il Lee

**Affiliations:** a ‘New-Senior’ Oriented Smart Health Care Education Center, Pukyong National University, Busan, 48513, Republic of Korea; b Department of Bioresources, University of Kashmir, Hazratbal, Srinagar, 190006, India; c Singapore-MIT Alliance for Research and Technology (SMART), Critical Analytics for Manufacturing Personalized-Medicine (CAMP), Create Way, 138602, Singapore; d Department of Physics, Indian Institute of Technology Delhi, New Delhi, 110016, India; e Department of Polymer-Nano Science and Technology, Jeonbuk National University, 567 Baekje-daero, Deokjin-gu, Jeonju-si, Jeollabuk-do, Republic of Korea; f Department of Chemistry, College of Science and Humanities in Al-Kharj, Prince Sattam Bin Abdulaziz University, Al-Kharj, 11942, Saudi Arabia; g Department of Pharmacology & Therapeutics, Roswell Park Cancer Institute, Buffalo, New York, 14263, United States; h Industry 4.0 Convergence Bionics Engineering, Pukyong National University, Busan, 48513, Republic of Korea; i Digital Healthcare Research Center, Institute of Information Technology and Convergence, Pukyong National University, Busan, 48513, Republic of Korea; j Division of Smart Healthcare, College of Information Technology and Convergence, Pukyong National University, Busan, 48513, Republic of Korea

**Keywords:** Hybrid nanostructure, ZnO nanorod, Vertically-aligned, Iron oxide nanoparticle, Non-enzymatic, Cholesterol biosensor

## Abstract

Developing highly sensitive and selective non-enzymatic electrochemical biosensors for disease biomarker detection has become challenging in healthcare applications. However, advances in material science are opening new avenues for creating more dependable biosensing technologies. In this context, the present work introduces a novel approach by engineering a hybrid structure of zinc oxide nanorod (ZnO NR) modified with iron oxide nanoparticle (Fe_2_O_3_ NP) on an FTO electrode. This Fe_2_O_3_ NP-ZnO NR hybrid material functions as a nanozyme, facilitating the catalysis of cholesterol and enabling the direct transfer of electrons to the fluorine-doped tin oxide (FTO) electrode, limiting the need for costly and traditional enzymes in the detection process. This innovative non-enzymatic cholesterol biosensor showcases remarkable sensitivity, registering at 642.8 μA/mMcm^2^ within a linear response range of up to 9.0 mM. It also exhibits a low detection limit (LOD) of ∼12.4 μM, ensuring its capability to detect minimal concentrations of cholesterol accurately. Moreover, the developed biosensor displays exceptional selectivity by effectively distinguishing cholesterol molecules from other interfering biological species, while exhibiting outstanding stability and reproducibility. Our findings indicate that the Fe_2_O_3_ NP-ZnO NR hybrid nanostructure on the FTO electrode holds promise for enhancing biosensor stability. Furthermore, the present device fabrication platform offers versatility, as it can be adapted with various enzymes or modified with different metal oxides, potentially broadening its applicability in a wide range of biomarkers detection.

## Highlights

• Successful growth of Fe_2_O_3_ NP modified ZnO NR on the FTO electrode.
• Fe_2_O_3_ NP-ZnO NR hybrid structure used as non-enzymatic electrochemical sensor for cholesterol detection.
• Non-enzymatic sensor demonstrates high sensitivity (642.8 μA/mMcm^2^) towards cholesterol detection.
• Exceptional selectivity against interfering species and reproducibility were obtained.

## Graphical abstract

**Figure:**
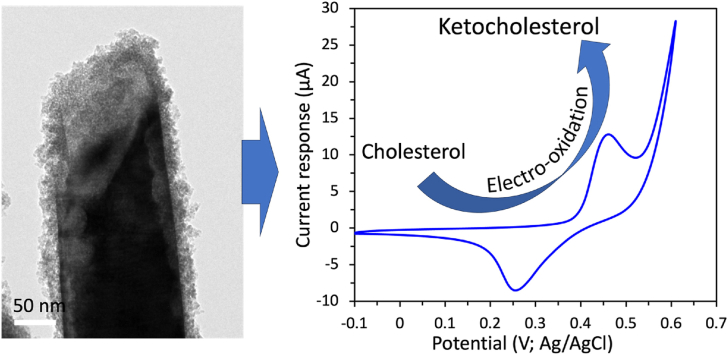

## 1 Introduction

Cholesterol monitoring is pivotal for cardiovascular health management, disease diagnosis, lifestyle adjustments, and medication regulation [1,2]. Abnormal high cholesterol levels, especially those surpassing the standard human serum range of 120–260 mg/dL, indicate potential health complications like heart disease, strokes, metabolic syndrome, and thyroid issues [2,3]. Thus, consistent cholesterol testing is essential for early diagnosis and effective treatment monitoring. Traditional methods for blood cholesterol measurement include high-performance liquid chromatography and spectrophotometry are known for their accuracy and reliability [[4], [5], [6]]. However, with the advent of electrochemical biosensors, the focus has shifted towards the development and application of innovative electrochemical devices [[7], [8], [9]].

Electrochemical-based cholesterol biosensors have become increasingly prominent in medical diagnostics and health monitoring [[10], [11], [12], [13]]. Their development represents a significant stride in medical diagnostics, providing a simpler, faster, and potentially more accessible technology to monitor cholesterol levels in real-time, thereby facilitating timely intervention and better health outcomes. Despite advanced capabilities in cholesterol detection, these biosensors face hurdles including stability concerns, vulnerability to external interference, and complexities in their fabrication process [[11], [12], [13]]. Overcoming these challenges is essential for their further advancement and broader application in managing cholesterol levels effectively. A noteworthy advancement in this area is the integration of nanomaterials, termed 'nanozymes', which serve as substitutes for traditional enzymes. This innovative approach significantly enhances biosensor stability, offers a pathway to streamline the manufacturing process, and reduces associated costs. The adoption of 'nanozymes' marks a significant stride towards enhancing the performance and accessibility of electrochemical cholesterol biosensors, paving the way for their increased utilization in healthcare settings.

Nanostructured metal oxides (MOx) are revolutionizing the field of electrochemical-based biosensors, both enzymatic and non-enzymatic, due to their high surface area [[14], [15], [16], [17], [18], [19], [20]]. This key feature allows for enhanced enzyme absorption or functionalization with additional metal oxide nanomaterials, thus significantly boosting biosensor performance [16,19]. Presently, there is a shift in focus towards hybrid nanostructures in sensing applications, which are favored for their superior synergetic composite properties [[21], [22], [23], [24]]. These hybrid nanostructures deliver improved performance from the amalgamation of various materials, which is otherwise unattainable by the single-material nanostructure [[25], [26], [27], [28], [29], [30], [31], [32], [33], [34], [35], [36], [37], [38], [39], [40]]. This advancement exemplifies progress in biosensor technology and opens new avenues for developing highly efficient and sensitive diagnostic tools, marking a significant leap forward in analytical capabilities and application potential in the electrochemical biosensing domain.

ZnO nanostructures have gained popularity in biosensing applications, particularly for non-enzymatic sensing, due to their ease of surface modification with metals or metal oxides [[25], [26], [27], [28], [29], [30], [31], [32], [33], [34], [35], [36], [37], [38]]. This adaptability makes ZnO nanostructures highly suitable for producing sensitive and selective biosensors. For instance, ZnO NR combined with copper oxide nanoparticles (CuO NPs) have been effectively used in electrochemical biosensors for glucose [25,26] and uric acid [27] detection. Similarly, ZnO NR-Fe_2_O_3_ NP hybrids have shown promise for glucose [28] and nitrite [29] sensing. Further, field-effect transistors (FETs) based on ZnO NR-Fe_2_O_3_ NP have been developed for the detection of glucose [30], cholesterol [31], potassium [32], and calcium [33]. Additionally, ZnO NR-NiO quantum dot (QD)-based FETs have been utilized for glucose sensing [34], while FET biosensors incorporating ZnO NR modified with Ag, Co, and MnO_2_ NPs have been explored for the detection of multiple ions, including phosphate, nitrate, and potassium [35]. These advancements highlight the broad applicability and efficiency of ZnO nanostructures in diverse biosensing contexts, showcasing their significant impact on the advancement of novel diagnostic tools.

In this study, we adopted a two-step hydrothermal process to fabricate Fe_2_O_3_ NP-decorated vertically aligned ZnO NR hybrid nanostructures on a conductive FTO electrode. Initially, the synthesis involved creating vertically aligned ZnO NR, which were subsequently modified with Fe_2_O_3_ NP through a dip-coating technique using an iron oxide precursor. A comprehensive examination of the electrochemical properties of the Fe_2_O_3_ NP-ZnO NR/FTO electrode and assessed its utility as a non-enzymatic biosensor. The biosensor's efficacy in cholesterol detection was investigated by utilizing amperometric analysis, it showcased notable sensitivity to cholesterol levels up to 9.0 mM. Further investigations were carried out to analyze other critical sensing parameters, such as the LOD, selectivity, stability, and reproducibility. The findings from this study suggest that the developed cholesterol biosensor presents a straightforward manufacturing process and maintains high stability and sensitivity, particularly for detecting low cholesterol concentrations.

## 2 Experimental details

### 2.1 Materials

The chemicals for the experiments were used directly as received without further processing. A range of reagents were procured from Sigma-Aldrich, including zinc nitrate hexahydrate (Zn(NO_3_)_2_·6H_2_O), cholesterol, iron (III) nitrate nonahydrate (Fe(NO_3_)_3_·9H_2_O), hexamethylenetetramine (HMTA), phosphate-buffered saline (PBS) solution (pH 7.4), and cholesterol oxidase (Streptomyces sp.), among others. Additionally, experiments involved other chemicals like D-(+)-glucose, uric acid (UA), lactic acid (LA), L-cysteine (L-cys), fructose, and dopamine (DA) to test the biosensor's selectivity. The preparation of all solutions and the materials synthesis were carried out using ultrapure water to ensure the highest quality and reliability of the experimental results.

### 2.2 ZnO NR and Fe_2_O_3_ NP-ZnO NR synthesis on FTO electrode

To construct a Fe_2_O_3_ NP-ZnO NR/FTO biosensor electrode, the FTO substrates were thoroughly cleaned using ethanol, water, and ultrasonication. This was followed by depositing a ZnO seed layer, approximately 50 nm thick, on the FTO substrates *via* radio frequency (RF) sputtering to facilitate the growth of highly-aligned ZnO NR. The nanorods were grown hydrothermally in a specially prepared solution of Zn(NO_3_)_2_·6H_2_O (40 mM) and HMTA (40 mM) in 100 mL ultrapure water with the seeded FTO substrate positioned upside-down and heated at 80 °C for 3 h. Post-growth, the substrates were rinsed to remove any impurities. Then, the vertically aligned ZnO NR surface was modified by synthesizing Fe_2_O_3_ NP on it using a simple dip-coating technique. The ZnO NR/FTO electrode was dipped in the solution containing 0.06g Fe(NO_3_)_3_·9H_2_O in 20 mL of water for 2 min [29]. The electrode was dried and annealed at 400 °C for 2 h. A thin layer of Nafion solution was applied to the surface of the Fe_2_O_3_ NP-ZnO NR/FTO biosensor electrode to enhance selectivity. The biosensors were stored at room temperature after fabrication.

### 2.3 Material characterization and sensing measurements

Morphologies of ZnO NR and Fe_2_O_3_ NP-ZnO NR were analyzed using a Hitachi S4700 field-emission scanning electron microscope (FESEM). Elemental analysis was conducted through energy dispersive spectroscopy (EDS) attached to the FESEM. The JEOL-JEM-2010 transmission electron microscope (TEM) was employed to verify the surface modification of ZnO NR with Fe_2_O_3_ NP. The crystal analysis of the ZnO NR and Fe_2_O_3_ NP-ZnO NR was performed using X-ray diffraction (XRD) with a Rigaku instrument. This analysis covered an angular range of 10–60° and was conducted at a scanning speed of 8° per minute. Additionally, the chemical composition and atomic chemical states in the Fe_2_O_3_ NP-ZnO NR were examined using the AXIS-NOVA X-ray photoelectron spectroscopy (XPS) system from Kratos Inc. This analysis employed a monochromatic Al Kα X-ray source for detailed surface characterization.

Electrochemical analyses were conducted using a 3-electrode system, comprising an Ag/AgCl reference, platinum counter, and Fe_2_O_3_ NP-ZnO NR/FTO as working electrode. These were connected to an electrochemical measurement system (PalmSens4, a compact potentiostat). Electrochemical impedance spectroscopy (EIS) was performed in a mixed 5 mM [Fe(CN)_6_]^3−/4−^ and 100 mM KCl solution, across a frequency range of 0.1 Hz–100 MHz. The obtained data were interpreted using a Randles equivalent circuit model, and all potentials were applied *via* an Ag/AgCl electrode.

### 2.4 Statistical analysis

The XRD, XPS, CV, EIS, and amperometric data were plotted using the scientific plotting software “Origin®“. For plotting the calibration curve, at least n = 3 independent amperometric response replicates against known cholesterol concentrations were performed for each experiment. The linear regression analysis is then used to determine the relationship between the sensor response and cholesterol concentration.

## 3 Results and discussion

### 3.1 Material characterization

**Fig. 1:**
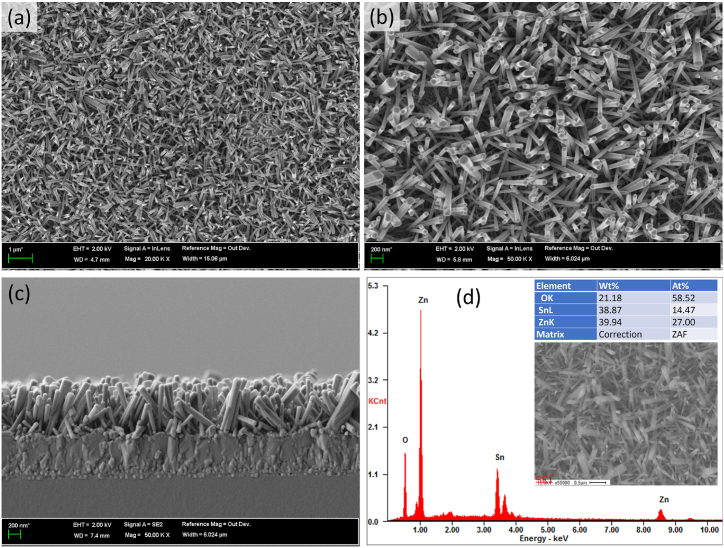
FESEM images showing planar view (a and b), cross-sectional view (c), and EDS pattern of the vertically aligned ZnO NR. Insets in d show the elements present and the FESEM image where EDS spectra were measured.

Fig. 1 presents the successful growth of ZnO NR on an FTO electrode, as depicted through FESEM images. These images detail the surface morphology at varying resolutions (Fig. 1a

and 1b) and provide a cross-sectional view (Fig. 1c), complemented by EDS surface analysis (Fig. 1d). The low-resolution FESEM image (Fig. 1a) clearly shows the uniform growth pattern of ZnO NR. The cross-sectional imagery highlights the vertical alignment of ZnO NR, revealing dimensions approximately 850 nm in length and 90 ± 10 nm in diameter (Fig. 1c). Moreover, the EDS analysis corroborates the purity of the elemental composition showing exclusively zinc and

oxygen peaks, indicating the absence of impurities (Fig. 1d). Additionally, the EDS spectrum reveals the presence of tin (Sn) element, originating from the FTO substrate. This comprehensive imaging and analysis affirm the successful and pure growth of ZnO NR on the FTO electrode, setting a solid foundation for subsequent steps for biosensor fabrication.

**Fig. 2:**
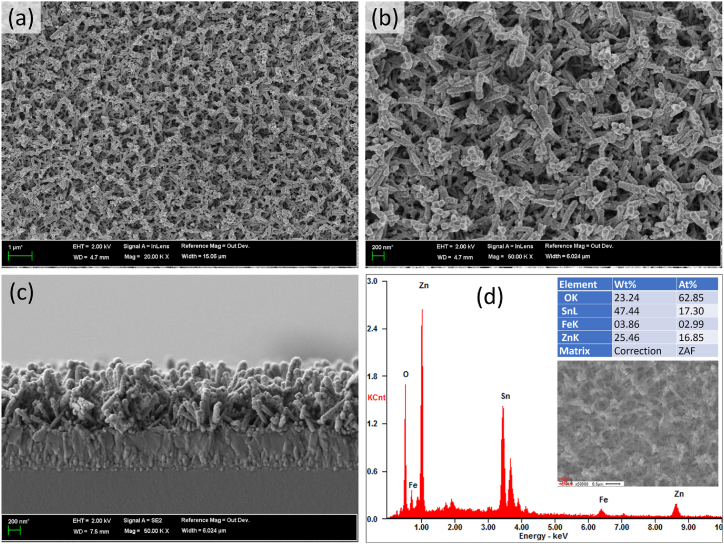
FESEM images showing planar view (a and b), cross-sectional view (c), and EDS pattern of the Fe_2_O_3_ NP modified vertically aligned ZnO NR. Insets in d show the elements present and the FESEM image where EDS spectra were measured.

Surface modification of ZnO NR is a pivotal process, as it can notably enhance the nanostructure's functionality by introducing new properties and improving catalytic performance. The integration of Fe_2_O_3_ NP with ZnO NR was thoroughly examined using FESEM as depicted in Fig. 2. The incorporation of Fe_2_O_3_ NP onto ZnO NR resulted in a discernibly rougher surface texture, signifying the successful attachment of nanoparticles, as shown in Fig. 2a–c. This textural change indicates effective surface engineering, aiming at the nanostructure's catalytic capabilities. Further, the EDS analysis confirmed the modification of ZnO NR with Fe_2_O_3_ NP, as evidenced by the clear peaks of zinc, oxygen and iron present in Fig. 2d. This verifies the successful modification and suggests potential enhancements in the nanostructure's catalytic activity and functional properties.

**Fig. 3:**
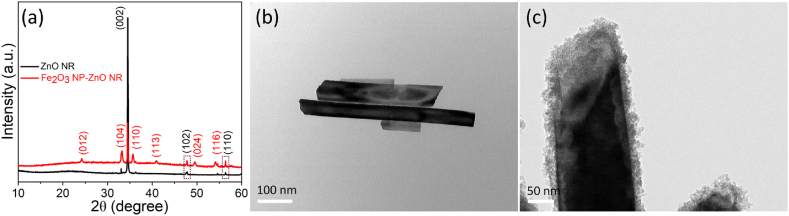
XRD patterns (a), TEM images of ZnO NR (b) and hybrid Fe_2_O_3_ NP-ZnO NR (c).

The phase-structure crystallinity of ZnO NR and Fe_2_O_3_ NP-ZnO NR were analyzed using XRD, as depicted in Fig. 3a. The diffraction peaks for the ZnO NR were well-matched with standard JCPDS card No.: 36–1451, ensuring their purity and confirming their structure [31]. However, additional peaks for ZnO NR modified with Fe_2_O_3_ NP indicated the hexagonal structure of Fe_2_O_3_ (JCPDS: 33–0664) [29]. This modification also led to the peak intensities change of ZnO NR XRD spectra. The sharpness of these diffraction peaks implies that both the ZnO NR and the Fe_2_O_3_ NP-ZnO NR possess a high degree of crystallinity. The absence of impurity peaks in the XRD patterns underscored the purity of the pristine ZnO NR and Fe_2_O_3_ NP-ZnO NR nanostructures. This was further corroborated by the TEM of the ZnO NR and the hybrid Fe_2_O_3_ NP-ZnO NR, as shown in Fig. 3b and c. The TEM images depicted the Fe_2_O_3_ NP on the ZnO.

NR, further confirming the observations made through FESEM analysis, which had previously indicated a rough surface texture post-Fe_2_O_3_ NP modification on the ZnO NR.

**Fig. 4:**
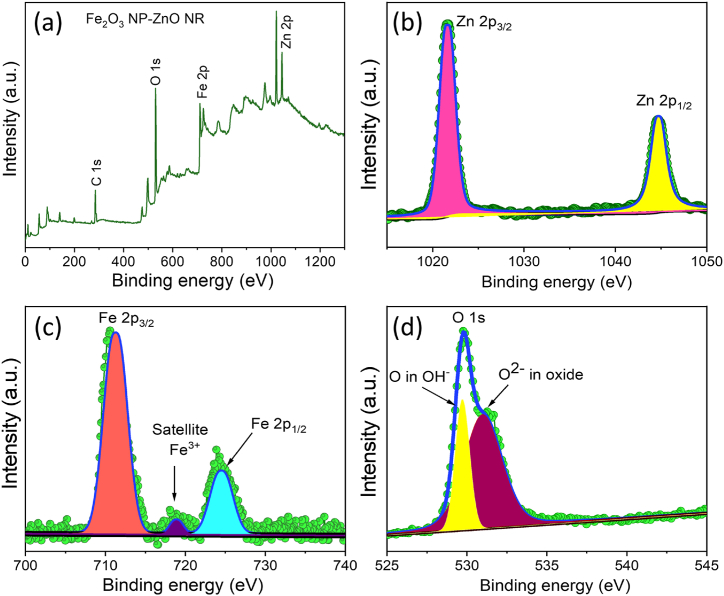
Survey spectrum of Fe_2_O_3_ NP-ZnO NR (a) and narrow scan spectra for Zn 2p (b), Fe 2p (c), and O 1s (d).

The compositional analysis of the Fe_2_O_3_ NP-modified ZnO NR hybrid was meticulously conducted using XPS, as depicted in Fig. 4. The XPS full scan confirmed the presence of the constituent elements of the hybrid nanostructures by pinpointing the specific binding energies of carbon (C 1s), oxygen (O 1s), zinc (Zn 2p), and iron (Fe 2p). Notably, the Zn 2p high-resolution spectrum exhibited two prominent peaks, aligning with the expected electronic configuration of

zinc (Fig. 4b). Similarly, the Fe 2p spectrum revealed binding energy peaks characteristic of Fe_2_O_3_, including a notable satellite peak that signifies the presence of Fe^3+^ ions, a marker of the oxidation state (Fig. 4c) [41,42]. Moreover, the O 1s spectrum was distinguished by two peaks, corresponding to oxygen atoms bonded within the crystal lattice to zinc and iron, respectively (Fig. 4d) [42]. This detailed XPS analysis conclusively demonstrated the successful modification of ZnO NRs with Fe_2_O_3_ NPs, highlighting the synthesised hybrid material's precise elemental composition and chemical state.

### 3.2 Electrochemical properties of modified electrodes

**Fig. 5:**
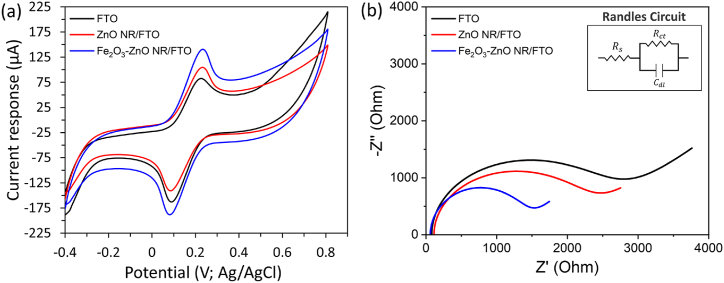
(a) CV responses of the (i) bare FTO, (ii) ZnO NR/FTO, (iii) Fe_2_O_3_ NP-ZnO NR/FTO in a mixture of 5 mM [Fe(CN)_6_]^3−/4−^ and 100 mM KCl solution, at a scan rate of 50 mV/s (b) EIS spectra of the (i) bare FTO, (ii) ZnO NR/FTO, (iii) Fe_2_O_3_-ZnO NR/FTO in a mixture of 5 mM [Fe(CN)_6_]^3−/4−^ and 100 mM KCl solution (frequency range = 10^−1^ to 10^5^ Hz, applied potential = 0.12 V.

The CV and EIS measurements were employed to assess the electrochemical characteristics of both bare and modified FTO electrodes, as illustrated in Fig. 5. The electrochemical behavior of the bare FTO, ZnO NR/FTO, and Fe_2_O_3_ NP-ZnO NR/FTO electrodes was examined in a solution containing 5 mM [Fe(CN)_6_]^3−/4−^ and 100 mM KCl, at a scan rate of 50 mV/s (Fig. 5a). Among these, the Fe_2_O_3_ NP-ZnO NR/FTO electrode exhibited the most superior current response. This enhanced performance can be attributed to the surface modification of the FTO electrode with the ZnO NR and Fe_2_O_3_ NP, which significantly improves the electron transfer efficiency [33]. The results clearly demonstrate how the strategic addition of ZnO NR and Fe_2_O_3_ NP to the FTO electrode surface plays a pivotal role in boosting its electrochemical properties, offering insights into the potential of such modifications in advancing electrochemical sensor technology.

Additionally, to confirm the above observation of current response change due to surface modification of FTO electrode. The EIS spectra of the bare FTO, ZnO NR/FTO, and Fe_2_O_3_ NP-ZnO NR/FTO were measured and curves were fitted using the Randles equivalent circuit. The fitted Nyquist diagrams are shown in Fig. 5b. The fitted impedance data reveals the change in charge transfer resistance (R_*ct*_) with ZnO NR and Fe_2_O_3_ NP modifications. The bare FTO electrode exhibited a high R_*ct*_ of ∼2750 Ω, as indicated by its large Nyquist semicircle. After growing ZnO NR on the FTO electrode, the semicircle size reduced and the R_*ct*_ decreased to ∼2360 Ω, showing an improved electron transfer rate. With the further addition of Fe_2_O_3_ NP, the optimal electron transfer rate was achieved with a much lower R_*ct*_ of ∼980 Ω. These findings highlight the effectiveness of Fe_2_O_3_ NP modification in facilitating efficient electron transfer on ZnO NR/FTO electrodes, underscoring the potential of these modified electrodes for advanced electrochemical studies and applications.

### 3.3 Electrochemical sensing of cholesterol at Fe_2_O_3_ NP-ZnO NR/FTO electrode

**Fig. 6:**
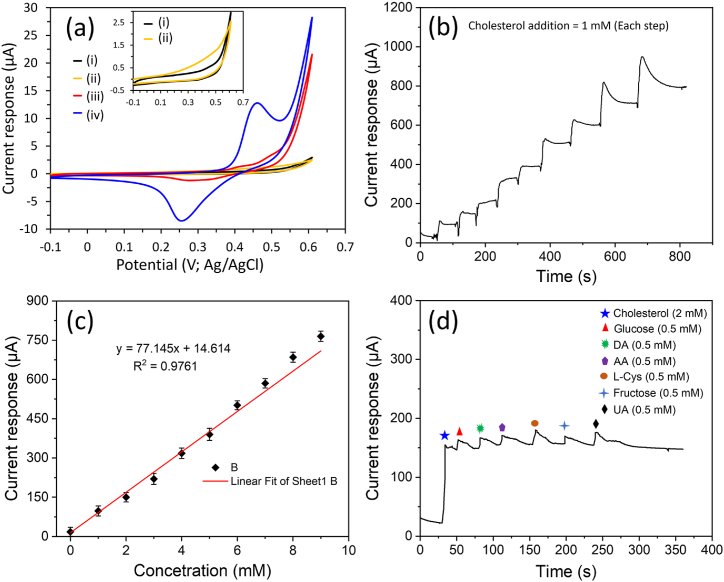
(a) CV responses of the ZnO NR/FTO and Fe_2_O_3_ NP-ZnO NR/FTO electrodes in the PBS buffer (curve (i) for ZnO NR/FTO and curve (ii) Fe_2_O_3_ NP-ZnO NR/FTO) and in the PBS buffer having 0.5 mM cholesterol (curve (iii) for ZnO NR/FTO and curve (iv) Fe_2_O_3_ NP-ZnO NR/FTO), (b) amperometric response of the Fe_2_O_3_ NP-ZnO NR/FTO electrode with increasing cholesterol concentration up to 9.0 mM, (c) current response *vs.* concentration calibration plot, and (d) interference study test in the presence of potential interfering substances.

Initially, the CV response of the ZnO NR/FTO and Fe_2_O_3_-ZnO NR/FTO electrodes were tested for their electrochemical properties in non-enzymatic cholesterol detection. The CV

response was recorded in the PBS buffer without and with 0.5 mM cholesterol (Fig. 6a). In the presence of cholesterol, the CV response indicated that the Fe_2_O_3_ NP modification on ZnO NR/FTO electrodes significantly enhanced the current response (as compared to ZnO NR/FTO electrode) due to better electrocatalytic activities towards cholesterol oxidation. This improvement is also attributed to the increased surface area provided by the vertically aligned ZnO NR and non-enzymatic catalytic property of Fe_2_O_3_ NP.

As reported earlier, during the electrochemical oxidation of the cholesterol, the hydroxyl group is converted into a carbonyl group, which releases a hydride or proton [43,44]. This process involves a forward scan generating a dehydrogenated intermediate, while the reverse scan reduces cholesterol. The Fe_2_O_3_ NP-ZnO NR/FTO electrodes adsorb oxygen (O_2_) and convert it to O_2_^−^ at the electrode surface. This reacts with electrons and water to produce hydrogen peroxide (H_2_O_2_) and hydroxyl radicals (.OH), which initiate cholesterol electrooxidation, resulting in ketocholesterol formation. The oxidation process transfers electrons through the electrode and generates current. The Fe_2_O_3_ NP modified on the ZnO NR surface enhances electrocatalytic activity, promoting cholesterol oxidation on the electrode. The possible non-enzymatic cholesterol electrooxidation is presented below [43].

(a) $${\mathrm{Fe}}_{2}O_{3}+O_{2}+e^{-}\to \;{\mathrm{Fe}}_{2}O_{3}+{O_{2}}^{-}$$

(b) $$O_{2}^{-}+2H_{2}O\;\to \;H_{2}O_{2}+2\;.\text{OH}$$

(c) $$O_{2}^{-}+H_{2}O_{2}\;\to \;.\text{OH}+{\text{OH}}^{-}+O_{2}$$

(d) .OH+Cholesterol→ketocholesterol

Further, the amperometric detection method investigated the analytical performance of the Fe_2_O_3_ NP-ZnO NR/FTO biosensor electrode. The amperometric response of the non-enzymatic Fe_2_O_3_ NP-ZnO NR/FTO biosensor electrode was measured with increasing cholesterol concentration up to 9.0 mM at the constant potential of 0.46 V (Fig. 6b). The cholesterol (1 mM) was added at each measurement step, which shows an increase in the current response after each addition. To calculate the performance of the non-enzymatic Fe_2_O_3_ NP-ZnO NR/FTO cholesterol biosensor, a calibration curve of the current response *vs.* cholesterol concentration was plotted (Fig. 6c). The biosensor showed a linear detection range of up to 9.0 mM cholesterol concentration. A linear regression equation (*y* (μA) = 77.145*x* ( × mM) + 14.614 with R^2^ of 0.9761) was obtained, where *x* is cholesterol concentration and *y* is the current response. Using the slope of the calibrated plot, the sensitivity of the Fe_2_O_3_ NP-ZnO NR/FTO biosensor was calculated to be 642.8 μA/mMcm^2^. Another critical parameter of the designed biosensor, i.e., LOD was calculated using signal-noise-ratio (S/N = 3). Table 1 presents a comparative performance of the Fe_2_O_3_ NP-ZnO NR/FTO biosensor in detecting cholesterol, highlighting its comparatively superior analytical performance over other biosensors reported in the literature [[45], [46], [47], [48], [49], [50]]. It demonstrates the straightforward preparation, potential for mass production, and the highly sensitive response attributed to vertically aligned Fe_2_O_3_ NP-modified ZnO NR that provides high catalytic sites for

**Table 1:** Shows a comparative Fe_2_O_3_ NP-ZnO NR/FTO biosensor performance with other reported cholesterol biosensors.

Biosensor electrode	Technique	Linear range (mM)	Sensitivity (μA/mMcm^2^)	LOD (μM)	Ref.
Fe_3_O_4_@SiO_2_/MWNT/GCE	Amperometric	0.01–4	–	5	[45]
ChOx/HSFe_2_O_3_/GCE	CV	0.05–8	9.94	50	[46]
ChOx/NS-CeO_2_/ITO	CV	0.25–10.25	5.98	250	[47]
ChOx/CoO(NPs)/GCE	Amperometric	0.0042–0.05	43.5	4.2	[48]
GOx/Chi-Ni(OH)_2_/GCE	Amperometric	0.45–10	0.276	49	[49]
ChOx/Cu-Pt-Bi/GCE	Amperometric	0.05–4	40.68	30	[50]
Fe_2_O_3_ NP-ZnO NR/FTO	Amperometric	0–9	642.8	∼12.4	This work

**Abbreviations:** Fe_3_O_4_, iron oxide; SiO_2_, silicon dioxide; MWNT, multi-walled carbon nanotube; GCE, glassy carbon electrode; ChOx, cholesterol Oxidase; HS, hollow spheres; CeO_2_, cerium oxide; ITO, indium-tin-oxide; CoOx, cobalt oxide; NPs, nanoparticles; Chi, chitosan; Ni(OH)_2_, nickel(II) hydroxide; NS, nano-structured; Cu, copper; Pt, platinum; Bi, bismuth.

cholesterol electrooxidation. This makes the Fe_2_O_3_ NP-ZnO NR material superior for selective and sensitive non-enzymatic biosensor fabrication.

### 3.4 Interference, stability, and reproducibility studies

The presence of interfering agents that oxidise at potentials similar to the target biomolecule can significantly hinder the selective detection of that particular analyte. Consequently, assessing the selectivity of a biosensor becomes a critical aspect of its evaluation. In this context, Fig. 6d presents the results of an interference study conducted for the Fe_2_O_3_ NP-ZnO NR/FTO cholesterol biosensor. The study examined the amperometric response of biosensors to various potential interfering substances, such as glucose, uric acid (UA), lactic acid (LA), L-cysteine (L-cys), fructose, and dopamine (DA), alongside a concentration of 2 mM cholesterol and 0.5 mM of each interferent. The outcome results indicated minimal interference from these compounds, showcasing the biosensor's high selectivity towards cholesterol. This finding underscores the capability of biosensor to accurately detect cholesterol even in other potentially interfering substances presence, highlighting its practical applicability in selective cholesterol monitoring.

The reproducibility of the non-enzymatic cholesterol biosensor, incorporating Fe_2_O_3_ NP and ZnO NR on an FTO electrode, was meticulously evaluated by fabricating and testing five identical biosensors. Each biosensor was subjected to CV analysis in a PBS solution containing 0.5 mM cholesterol. Remarkably, all five biosensors exhibited nearly identical current responses, with a relative standard deviation (RSD) of approximately 5.5 %, indicating excellent fabrication consistency. Furthermore, the storage stability of the biosensor was tested by keeping them at room temperature and periodically measuring their response every seven days. Impressively, after eight weeks, the biosensors retained about 92.8 % of their initial current response. This exceptional level of stability can be attributed to the direct growth technique used for ZnO NR on the FTO electrode, significantly bolstering the mechanical robustness of the biosensor. Such findings highlight the biosensor's reliable performance and long-term usability, making it a promising tool for cholesterol monitoring.

## 4 Conclusion

In this study, we have successfully developed a highly sensitive, non-enzymatic cholesterol biosensor by synthesizing vertically aligned ZnO NR on an FTO electrode and subsequently modifying these NR with Fe_2_O_3_ NP. A low-temperature hydrothermal synthesis approach was utilized for the electrode fabrication, where the ZnO NR were directly and vertically-grown on the FTO electrode. This structural arrangement, especially after the Fe_2_O_3_ NP modification, significantly expands the surface area available for biochemical interactions, thereby enhancing the electrochemical detection capabilities for cholesterol. The amperometric analysis of the Fe_2_O_3_ NP-ZnO NR/FTO biosensor demonstrated its outstanding sensitivity, broad response range, and remarkable stability. These exceptional attributes are primarily attributed to the Fe_2_O_3_ NP-modified ZnO NR nanostructures, which offer abundant catalytic sites for the electrooxidation of cholesterol alongside a conductive pathway for efficient electron transport. This innovative biosensor design ensures high performance in cholesterol detection and opens new avenues for developing other non-enzymatic biosensors leveraging similar nanostructured materials.

## Data availability statement

Data will be made available on request.

## CRediT authorship contribution statement

**Rafiq Ahmad:** Writing – review & editing, Writing – original draft, Methodology, Investigation, Formal analysis, Data curation, Conceptualization. **Kiesar Sideeq Bhat:** Writing – review & editing, Methodology, Investigation, Data curation. **Vandana Nagal:** Writing – review & editing, Methodology, Formal analysis. **Umesh T. Nakate:** Writing – review & editing, Methodology, Formal analysis, Data curation. **Akil Ahmad:** Writing – review & editing, Visualization, Validation, Software, Methodology, Formal analysis. **Mohammed B. Alshammari:** Writing – review & editing, Resources, Funding acquisition. **Shamshad Alam:** Writing – review & editing, Methodology. **Byeong-Il Lee:** Writing – review & editing, Supervision.

## Declaration of competing interest

The authors declare that they have no known competing financial interests or personal relationships that could have appeared to influence the work reported in this paper.
